# When are puppies receptive to emotion-induced human chemosignals? The cases of fear and happiness

**DOI:** 10.1007/s10071-023-01771-4

**Published:** 2023-04-03

**Authors:** Biagio D’Aniello, Claudia Pinelli, Anna Scandurra, Alfredo Di Lucrezia, Massimo Aria, Gün R. Semin

**Affiliations:** 1 Department of Biology, University of Naples Federico II, Via Cinthia, 80126 Naples, Italy; 2 Department of Environmental, Biological and Pharmaceutical Sciences & Technologies, University of Campania “Luigi Vanvitelli”, 81100 Caserta, Italy; 3 Department of Economics and Statistics, University of Naples Federico II, Via Cinthia, 80126 Naples, Italy; 4 William James Center for Research, ISPA-Instituto Universitario, 1149-041 Lisbon, Portugal; 5 Faculty of Social and Behavioral Sciences, Utrecht University, Utrecht, Netherlands

**Keywords:** Dogs, Human chemosignals, Emotions, Animal cognition

## Abstract

We report an observational, double-blind, experimental study that examines the effects of human emotional odors on puppies between 3 and 6 months and adult dogs (one year and upwards). Both groups were exposed to control, human fear, and happiness odors in a between subjects’ design. The duration of all behaviors directed to the apparatus, the door, the owner, a stranger, and stress behaviors was recorded. A discriminant analysis showed that the fear odor activates consistent behavior patterns for both puppies and adult dogs. However, no behavioral differences between the control and happiness odor conditions were found in the case of puppies. In contrast, adult dogs reveal distinctive patterns for all three odor conditions. We argue that responses to human fear chemosignals systematically influence the behaviors displayed by puppies and adult dogs, which *could* be genetically prefigured. In contrast, the effects of happiness odors constitute cues that require learning during early socialization processes, which yield consistent patterns only in adulthood.

## Introduction

One of the oldest and most widely used media for communication is chemosignals. It has been demonstrated that plants (Heil and Karban 2010) and bacteria (Taga and Bassler 2003) rely on communication via chemosignals. The information chemosignals contain is very rich and has been extensively examined. Indeed, various studies have revealed systematically that human chemosignals mediate neural, cognitive, and behavioral social processes (e.g., de Groot et al. 2017; Pause 2017; Semin and Groot 2013; Stevenson 2010). A domain that has commanded considerable attention is the transfer of emotional information, namely the information that human body odors (BOs) emitted during an emotional state (e.g., fear, happiness, disgust) by a donor (sender) and how these BOs affect a recipient (de Groot et al. 2017; Pause 2017). The recent interest in intraspecies communication has inspired pioneering work on interspecies communication. It has been shown that human emotional chemosignals shape the behaviors of other species, particularly dogs (D’Aniello et al. 2018, 2021; Semin et al. 2019; Siniscalchi et al. 2016), mice, cows (Destrez et al. 2021), and horses (Lanatà et al. 2018; Sabiniewicz et al. 2020).

Reading human emotional responses has an important adaptive value for dogs, allowing them to pick up information about environmental novelties and regulate their behavior accordingly in social referencing (Merola et al. 2012, 2013). The communicative link between humans and dogs has primarily focused on the visual and acoustic systems (D’Aniello et al. 2016; Scandurra et al. 2017), which can mediate emotional responses (Albuquerque et al. 2016). However, dogs are known to have extraordinary olfactory abilities, and their olfactory system is well known to be a significant contributor to the regulation of their social relations (Miklósi 2007; Thesen et al. 1993). While interspecies research, particularly between humans and dogs, has made great strides in recent years, the evidence about the determinate or indeterminate nature of the chemosignal effects (see for a general case Nielsen et al. 2015; Wyatt 2010) in the case of interspecies effects of chemosignals has remained an open question (see also Stowers and Marton 2005).

The issue we address in the research reported here examines whether the interspecies transfer of emotional information via fear vs. happiness chemosignals yields consistent behavior patterns across puppies and adult dogs.

To understand possible differences in the behavioral outcomes of exposure to fear and happiness, we must consider the features of owner-puppy interactions where the owner (or other household inhabitants) emits such chemosignals. We must also consider the possibility of evolutionarily conserved chemosignals. However, while an observational study is unlikely to provide inconvertible proof of a preconfigured behavioral determination of responses to humans’ fear and happy body odors, the constraints existing within such a puppy-human interaction could possibly narrow down the inferential potential of such an observational study.

The first question is how likely the owners will experience fear or happiness in their puppies' presence. In general, puppies are a source of happiness, and acquiring a puppy alone is likely a source of joy. Such a context allows puppies to associate happiness chemosignals with positive experiences. Otherwise, owners are more unlikely to experience fear while interacting with their puppies. One could surmise that, if anything, their socialization history would likely expose puppies to human happiness odors rather than fear odors. If this argument is correct and there is a learning effect for both chemosignals, one would expect an asymmetry in behavioral patterns of exposure to fear and happiness in human odors in favor of happiness.

However, it can be argued that happiness as a positive emotion may not occupy the same evolutionary ‘urgency’ as negative emotions, such as fear since the adaptive significance of fear appears to be self-evident. The assumption that happiness does not carry as much *“evolutionary salience”* as, for instance, fear is an argument (see Zhou and Chen 2009) suggesting that puppies are unlikely to display adult behavior patterns in response to happiness chemosignals. In this view, the behavioral responses to happiness chemosignals, particularly from other species, should be less likely to be inherited from our mammalian ancestors.

Another line of argument would suggest the opposite of the prominence of happiness-related behavior patterns being manifested. Suppose it is the case that certain affective states have highly adaptive value over the evolutionary course of history. In that case, one might expect puppies and adult dogs to display the same behavioral patterns when exposed to fear chemosignals. As has already been proposed by Darwin (Darwin 1872), fear is arguably an inner mental state inherited from our mammalian ancestors and is manifested behaviorally. Such behavioral responses emerge in biologically or socially challenging situations and can be seen across animal species from which we regularly infer fear (Mobbs et al. 2019). Furthermore, fear circuits are conserved in mammal brains (LeDoux 2012). This could mean that the chemical composition of human fear odors can constitute an evolutionarily conserved chemosignal. The argument that reactions to fear odor are evolutionarily conserved would tip the balance in favor of puppies displaying behavior patterns comparable to those of adults. Notably, an *observational* study revealing that puppies display adult behavior patterns when exposed to fear odors but not happiness odors may not support conclusively an innate fear response. Indeed, one also has to entertain the possibility of a biological preparedness for fast learning due to the salience of the fear responses.

Another plausible scenario is that puppies do not reveal any of the systematic responses that the two types of chemosignals activate, suggesting that the regular and robust patterns observed with adult dogs (D’Aniello et al. 2018) are the result of an extended socialization process. The opportunity to learn from humans during ontogenesis and thus shape (D’Aniello et al. 2015; Scandurra et al. 2015) and improve social-communicative skills, including chemosignal sensitivity, could be the result of the proximity between puppies and their owners (D’Aniello et al. 2016, 2017). Thus, dogs’ repeated interaction with humans can lead them to associatively learn the type of human chemosignals and the specific contexts in which they are emitted.

To examine the alternative outcomes of how adult dogs’ responses to human emotion odors of fear and happiness evolve, we designed a study in which we first extracted human fear and happiness odors. Then, we systematically examined the behavior of puppies younger than six months when exposed to human fear and happiness chemosignals, comparing these results with a sample of adult dogs.

## Materials and methods

### Odor collection

Odor collection was performed as reported in our earlier papers (D’Aniello et al. 2018, 2021). Heterosexual male donors were students at ISPA University, Lisbon (average age 21 years) (de Groot et al. 2012, 2015) who watched 25-min fear or happiness-inducing videos in two sessions separated by a week. They were asked to follow a strict protocol 2 days before the sweat donation: smoking, alcohol, odorous food, and excessive exercise were prohibited. Furthermore, donors had to use scent-free personal care products and were provided with odor-free detergents. The sweat was collected using sterile absorbent compresses (Cutisorb, BSN Medical, Hamburg, Germany) from both armpits. Sweat pads were stored at − 22 °C until they were transferred to the Italian laboratory on dry ice. They were stored in a − 80 °C freezer until they were used. The pads of four individuals were cut into four pieces to rule out interindividual differences in body odor and matched in the apparatus, thereby creating a pooled sample (Mitro et al. 2012). The ethics committee approved all the procedures for the sweat collection of the host institution. They were conducted under the standards of the American Psychological Association and the guidelines of the Declaration of Helsinki.

### Subjects

Volunteers with their dogs were recruited through personal contacts and the internet. All dogs lived in a household with at least two people and, although some subjects had free access to the garden, were kept indoors at night and most of the day. Some dogs had to be excluded because of fear-related problems before testing. Some testing sessions had to be interrupted because the dogs displayed destructive behaviors toward the apparatus or because the owners did not comply with the instructions by interacting with the dog during the testing. The final database included 141 pet dogs: 61 puppies 3–6 months old (26 females, 4.6 ± 1.1; 35 males, 4.8 ± 1.0); 80 adults, one year and upwards (39 females, 3.3 ± 4.8; 41 males 3.5 ± 2.2). We randomly allocated dogs in both major groups (puppy and adult) to one of the three odor conditions balancing developmental stage and sex. Therefore, 51 dogs were in the fear condition (9 female puppies; 12 male puppies; 15 female adults; 15 male adults); 46 dogs were in the happiness condition (8 female puppies; 12 male puppies; 13 female adults; 13 male adults); 44 dogs were used for the control condition (9 female puppies; 11 male puppies; 11 female adults; 13 male adults). The breed was not balanced since 76% of our samples consisted of Labrador and Golden Retrievers (68 Labrador and 39 Golden Retrievers). The other breeds included 7 Mongrels, 3 Irish Setters, 3 Border Collies, and 21 different breeds never represented more than 2 times. About 20% of dogs were neutered and distributed equally across the conditions, whereby breeds and reproductive stages were not controlled.

### Experimental setting

The study was conducted as reported in our previous papers (D’Aniello et al. 2018, 2021) at the University of Naples Federico II (Naples) in a 4 × 3 m room unknown to the dogs. The room was set up with two chairs in opposite corners (one for the owner and the stranger) and a water bowl in another corner. Three different female assistants took the stranger role. They were not in their menstrual period. The apparatus was positioned in the center of the room. The apparatus consisted of a 39.5 × 30 cm wooden board with a semitransparent plastic container fixed at the center. The sweat samples were placed in a container with a circular hole (diameter of 3 cm) on the lid. While allowing dogs to engage in the olfactory exploration of the contents, this method prevented the dog from contaminating the substances by direct contact. A separate apparatus was used for each condition to avoid chemosignal contamination. At the end of each test, the bowl, the apparatus, and the room were washed and cleaned. The room temperature was set to 24 °C and was constant across the experimental conditions. All the tests were recorded through a closed-circuit television system with 4 cameras.

### Experimental procedure

Before the testing session started, the owner was informed about the procedure without disclosing the goal. The dogs were left free outside the room where the trials took place for about 5–10 min. During this period, the laboratory staff limited their interaction with the dogs but were friendly whenever a dog approached one of the experimenters. Since it was planned to record drinking as a stress behavior, dogs were allowed to drink ad libitum before the test. The owner with the dog entered the experimental room, where the stranger (experimenter, E1) was seated. The dog was left free to familiarize itself with the room. After 1 min of familiarization, the owner was asked to hold the dog close to the chair to allow a second experimenter (E2) to enter the room and fix the apparatus at the center. As soon as E2 left the room and closed the door, the owner released the dog for the testing procedure lasting 2 min. Both the E1 and owner were instructed not to interact with the dog (they were given two magazines to avoid eye contact with the dog) during the test (even if solicited by the dog). They were blind to the condition they were in. Each dog was allocated randomly to only one condition: fear, happiness, or control (unused pads). At the end of each test, the samples were frozen again and reused no more than 4 times.

### Behavioral parameters

The duration of all behaviors related to specific targets (i.e., owner, stranger, door, and apparatus) were recorded, including the stressful behaviors. The dog’s approach was recorded when the dog was moving toward the target. Physical contact includes explorative behaviors, such as sniffing (at a distance not more than approximately 20 cm). Furthermore, physical interaction with the muzzle or legs, licking, and jumping up the target were also included. The gazing behavior at the target was recorded when the dog was in a stationary position. Gazing behavior toward the people was recorded when directed to the face of the subjects. The stressful behaviors included mouth licking (the dog licks its lips or nose), locomotion (dog walking, pacing, or running around without a clear target or exploratory intent), shaking off, scratching, yawning, barking, yapping, panting, drinking water.

All behaviors related to the apparatus, the door, and the people, such as approaching, interacting, and gazing, were grouped, including the stress signals in the behavioral categories: owner-directed behaviors, stranger-directed behaviors, apparatus-directed behaviors, door-directed behaviors, and stressful behaviors (Table 1). The *duration* of each behavior of puppies was recorded using Solomon Coder® beta 16.06.26 (ELTE TTK, Hungary). Two raters blind to the conditions independently coded the behavior *duration* of each ethological behavior for 15% of the videos (i.e. 21 videos). For each ethological category, a Pearson correlation between the two coders was computed, namely: owner-directed behaviors, *r* = 0.991, *p* < 0.001; stranger-directed behaviors, *r* = 0.986, *p* < 0.001; apparatus-directed behaviors, *r* = 0.912, *p* < 0.001; door-directed behaviors, *r* = 0.994, *p* < 0.001; stressful behaviors, *α* = 0.974, *p* < 0.001. In all cases, the means were not statistically different. The subsequent statistical analyses were based on the data from coder 1.

**Table 1:** The ethogram adopted

Categories	Behaviors	Description
Owner-directed behaviors	Approach owner	The dog approaches and is clearly oriented toward the owner
	Interaction with owner	The dog engages in physical contact with or sniffing the owner
	Gazing at owner	The dog looks at the owner's face from a stationary position
Stranger-directed behaviors	Approach stranger	The dog approaches and is clearly oriented toward the stranger
	Interaction with stranger	The dog engages in physical contact with or sniffs the stranger
	Gazing at stranger	The dog looks at the stranger's face from a stationary position
Apparatus-directed behaviors	Approach apparatus	The dog approaches and is clearly oriented toward the apparatus
	Interaction with apparatus	The dog engages in physical contact or sniffs the apparatus
	Gazing at apparatus	The dog looks at the apparatus from a stationary position
Door-directed behaviors	Approach door	The dog approaches and is clearly oriented toward the door
	Interaction with door	The dog engages in physical contact or sniffs the door
	Gazing at door	The dog looks at the door from a stationary position
Stressful behaviors	All behaviors indicating a stressful situation	Mouth licking (the dog licks its lips or nose), locomotion (dog walking, pacing, or running around without a clear target), shaking off, scratching, yawning, barking, yapping, panting, drinking water

### Data analysis

To demonstrate response differences as a function of sweat sampled under different conditions, we adopted a linear discriminant analysis (LDA) to examine how well the measured variables (i.e., behavioral categories) would predict the specific odor condition for each dog. We used a multivariate technique, discriminant analysis, to separate two groups of observations based on the observational variables measured on each adult dog and puppy samples to find the contribution of each variable in separating the groups. For comparative purposes, the analysis was run separately for adults and puppies. Then, given the significant results for the functions of the LDA, we compared the scores obtained from the two functions with the Kruskal–Wallis test and Mann–Whitney posthoc tests, which were Bonferroni corrected. All analyses were conducted in SPSS (SPSS Statistics, version 27; IBM Corp., Armonk, NY, USA). Data were first tested for normality with the Kolmogorov–Smirnov test, revealing that 90% of the data followed a normal distribution. However, there were deviations from normality in certain conditions, specifically in the case of Apparatus-directed behaviors for adults in the control (*p* < 0.001) and happiness (*p* = 0.01) conditions and for the stressful behaviors in puppies in the fear condition (*p* < 0.01). Despite these deviations from normality, the decision was made to proceed with discriminant analysis based on a previous demonstration by Pohar et al. (2004) that suggested that discriminant analysis can still be a valid tool even when normality assumptions are not violated.

## Results

**Fig. 1:**
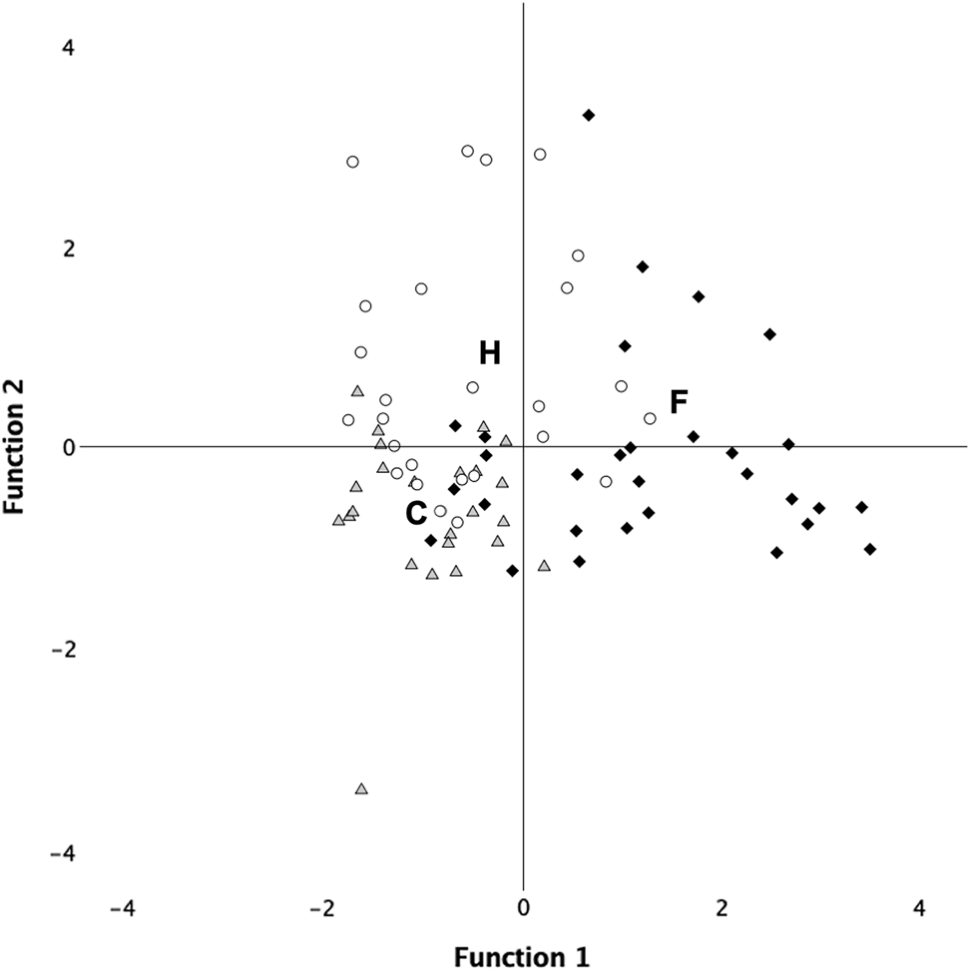
Graphical representation for the LDA plot for—adult conditions. The clusters are represented by gray triangles (Control), white circles (Happiness), and black rhombi (Fear), with centroids denoted by their corresponding letters (C, H, and F). The spatial distribution of Fear (F) was significantly different from both Control (C) and Happiness (H), while the spatial distribution of Happiness (H) and Control (C) was not statistically different. C (Control), H (Happiness), and F (Fear) represent the centroids of the clusters: gray triangles (control), white circles (happiness), and black rhombi (fear)

Descriptive data for the duration of the behavioral categories in the three conditions of puppies and adult dogs are reported in Table 2. The discriminant analysis (LDA) revealed two significant functions for adults (function 1: χ^2^ = 71.78, *p* < 0.001; function 2: χ^2^ = 20.50, *p* < 0.001). The scores obtained by function 1 significantly discriminated the three conditions (H = 37.05, *p* < 0.001). Post-hoc tests showed that the spatial distribution in the case of fear (Fig. 1) was significantly different from both the control (U = − 35.94, *p* < 0.001) and from the happiness (U = − 28.57, *p* < 0.001), while the spatial distribution of the happiness and control was not significantly different (U = − 7.37, *p* = 0.79). Significant loadings for function 1 were owner-directed behaviors, door-directed behaviors and stress. Significant loadings for function 2 were positive for stranger-directed behaviors and negative for apparatus-directed behaviors (Table 3).

**Table 2:** Descriptive data for the duration of the behavioral categories in the three conditions of puppies and adult dogs

Condition			Mean	Dev. st
Control	Adults	OWNER_D	9,58	8,03
		STRANGER_D	7,73	6,83
		APPARATUS_D	19,54	22,90
		DOOR_D	9,31	10,87
		STRESS_D	10,35	10,20
		OWNER_F	4,17	2,68
		STRANGER_F	2,42	1,74
		APPARATUS_F	4,33	1,66
		DOOR_F	3,38	3,66
		STRESS_F	3,38	2,84
	Puppies	OWNER_D	2,38	2,16
		STRANGER_D	5,96	6,84
		APPARATUS_D	15,67	12,53
		DOOR_D	2,25	4,23
		STRESS_D	4,07	5,01
		OWNER_F	1,40	1,23
		STRANGER_F	2,05	1,76
		APPARATUS_F	4,60	2,23
		DOOR_F	0,85	0,93
		STRESS_F	1,25	1,52
FEAR	Adults	OWNER_D	24,20	16,33
		STRANGER_D	11,70	10,62
		APPARATUS_D	12,77	8,83
		DOOR_D	18,87	17,86
		STRESS_D	53,75	26,43
		OWNER_F	8,17	4,59
		STRANGER_F	4,17	2,25
		APPARATUS_F	5,00	2,15
		DOOR_F	7,10	5,03
		STRESS_F	11,63	6,52
	Puppies	OWNER_D	7,75	7,33
		STRANGER_D	5,10	5,17
		APPARATUS_D	14,42	9,60
		DOOR_D	7,55	7,89
		STRESS_D	8,12	6,64
		OWNER_F	3,62	2,84
		STRANGER_F	2,62	2,11
		APPARATUS_F	5,81	1,86
		DOOR_F	3,81	3,53
		STRESS_F	3,76	2,10
Happines**s**	Adults	OWNER_D	9,02	7,29
		STRANGER_D	21,69	16,06
		APPARATUS_D	12,60	9,32
		DOOR_D	8,67	7,68
		STRESS_D	28,37	23,69
		OWNER_F	4,42	3,69
		STRANGER_F	4,73	2,31
		APPARATUS_F	4,12	1,63
		DOOR_F	3,35	2,91
		STRESS_F	7,27	6,35
	Puppies	OWNER_D	3,66	4,22
		STRANGER_D	6,49	6,93
		APPARATUS_D	14,03	9,81
		DOOR_D	5,02	5,03
		STRESS_D	3,03	4,34
		OWNER_F	2,25	2,53
		STRANGER_F	2,35	1,53
		APPARATUS_F	5,10	2,95
		DOOR_F	2,30	1,87
		STRESS_F	1,55	1,36

**Table 3:** Correlation with discriminant functions. Asterisks indicate significant loadings for each discriminant function. Data for Function 2 in puppies are omitted, as no significant variables were revealed

		Function 1	Function 2
0.378	Stress	0.818*	
	Owner-directed behaviors	0.624*	0.204
	Door-directed behaviors	0.365*	− 0.134
	Stranger-directed behaviors	− 0.640	0.874*
	Apparatus-directed behaviors	− 0.136	− 0.308*
Puppies	Owner-directed behaviors	0.566*	
	Stress	0.476	
	Door-directed behaviors	0.424	
	Apparatus-directed behaviors	− 0.037	
	Stranger-directed behaviors	− 0.096	

**Fig. 2:**
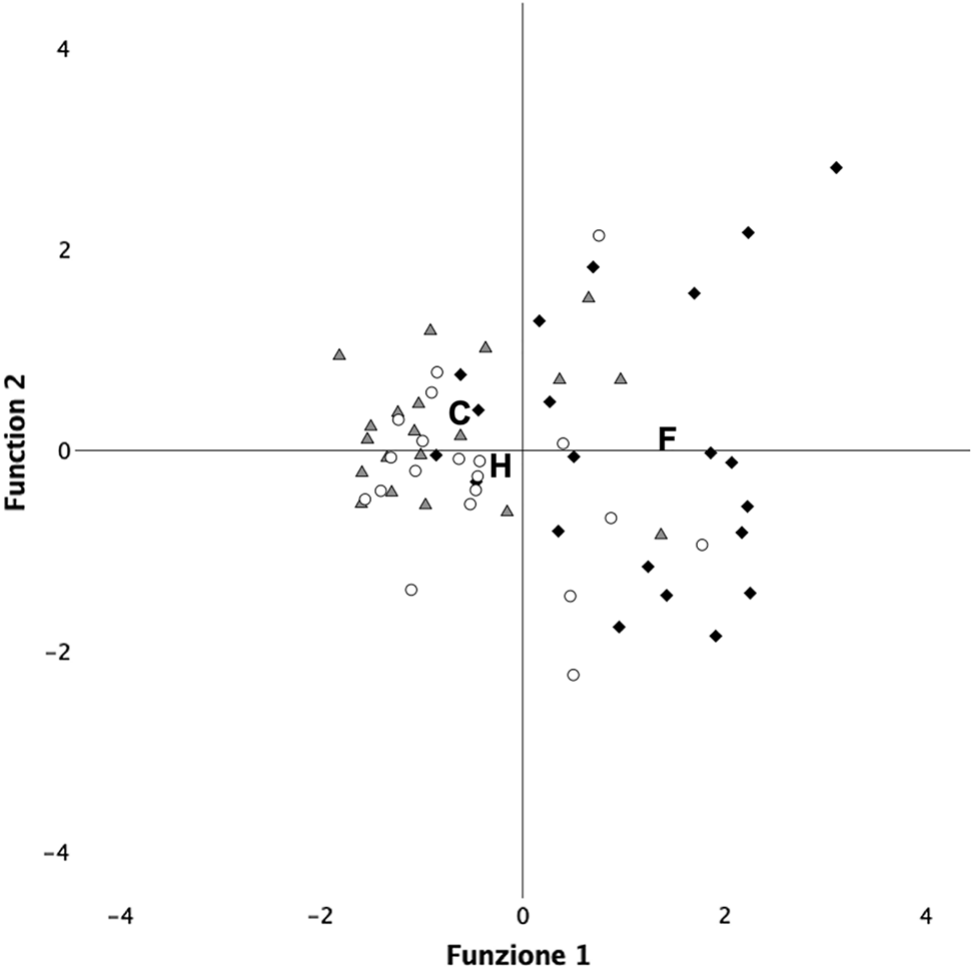
Graphical representation for LDA plot for – puppy conditions (based on only the significant function 1). The clusters are represented by gray triangles (Control), white circles (Happiness), and black rhombi (Fear), with centroids denoted by their corresponding letters (C, H, and F). C (Control), H (Happiness), and F (Fear) represent the centroids of the clusters: gray triangles (control), white circles (happiness), and black rhombi (fear). The spatial distribution of Fear (F) was significantly different from both Control (C) and Happiness (H). However, the spatial distribution of Happiness (H) and Control (C) was not significantly different

For puppies, the LDA revealed only one significant function (function 1: χ^2^ = 31.01; *p* < 0.001; function 2: χ^2^ = 2.21, *p* = 0.70). The scores for function 1 discriminated significantly between the three conditions (H = 23.36, *p* < 0.001). Post-hoc tests showed that this was due to the fear spatial distribution (Fig. 2) being significantly different from both the control (U = − 25.61, *p* < 0.001) and the happiness (U = − 19.41, *p* = 0.001). However, the spatial distribution of the happiness and control was not significantly different (U = − 6.20, *p* = 0.81). Significant loading for function 1 was owner-directed behaviors (Table 3).

When we now turn to the a priori and predicted instances, the classification matrix for adults based on a priori and predicted instances (Table 4) shows that 87.5% of the cases were in the predicted cell in the control condition, 73.3% in the predicted cell for the fear condition, and 53.8% in the happiness condition. For puppies, 70% of the cases were in the predicted cell in the control condition, 71.4% in the fear condition, and only 40% in the happiness condition.

**Table 4:** A priori and predicted classification overlap LDA

Status		Conditions	C	F	H
Adults	Count	C	21	0	3
		F	5	22	3
		H	9	3	14
	%	C	87,5	0.0	12.5
		F	16.7	73,3	10.0
		H	34.6	11.5	53,8
Puppies	Count	C	14	4	2
		F	4	15	2
		H	8	4	8
	%	C	70.0	20.0	10.0
		F	19.0	71.4	9.5
		H	40.0	20.0	40.0

## Discussion and conclusions

Our analyses reveal consistency with our earlier results for adult dogs (D’Aniello et al. 2018). However, the puppy results in the happiness condition showed a different pattern. The first discriminant function for puppies and adults is similar. Both show a significant loading for owner-directed behaviors. In the case of adults, stress behaviors and door-directed behaviors (indicating an attempt to escape the experimental room) are relatively prominent, as marked by the positive loadings.

Additionally, for the adult dogs, the second discriminant function is loaded by ‘stranger-directed behaviors’ (positively) and ‘apparatus-directed behaviors’ (negatively). This highlights affiliative behaviors toward the stranger activated by the positive emotion chemosignals of happiness. It is noteworthy that the fear barycenter of adults and puppies occupy the same position in the space (compare Figs. 1 and 2), further supporting the view of similar fear responses in younger and older dogs. Remarkably, the distinctive patterns that can be gleaned from the discriminant functions suggest that the proximity to the owner is important in the case of fear chemosignals for both adults and puppies. Proximity seeking is a typical attachment response (Prato-Previde et al. 2003; Scandurra et al. 2016; Topál et al. 1998), inducing dogs to return toward the owner in the presence of perceived threats (safe-haven effect, see Gácsi et al. 2013). Under such circumstances, dogs look for visual and physical comfort from their owners. Social smells activate the caudate nucleus in dogs (Berns et al. 2015), which has a relevant role in positive expectations in many species (Berns et al. 2012, 2013; Knutson et al. 2001; Montague and Berns 2002; Schultz et al. 1997), including social rewards (Izuma et al. 2008; Rilling et al. 2002). Notably, the dog’s caudate nucleus is activated more strongly when it is exposed to the body odor of a familiar human compared to odors from a familiar or a strange dog and an unfamiliar human (Berns et al. 2015), suggesting a positive emotional response to the odor of a familiar human (Bekoff 2007; Panksep 2004). Proximity to the owner enhances the accessibility of the owner’s odors, which can have beneficial effects in reducing the unpleasant emotion triggered by the fear of emotional chemosignals. Indeed, we know from research on humans that exposure to a caregiver’s body odor has a calming effect (Granqvist et al. 2019; Hofer and Chen 2020).

In the case of the human happiness condition, the puppies’ results largely overlap with the control condition. Happiness odors do not produce a behaviorally distinct repertoire as it does for adults. The most likely conclusion is that the effects of happiness-related behaviors are ontogenetically acquired over an extended period of social interaction between puppies and their human family. If happiness odors were genetically preconfigured and responsible for acquiring a behavioral repertoire, then the behavioral repertoire for happiness would have emerged earlier and be stable across development. However, this was not the case.

The evidence on happiness chemosignals from humans (de Groot et al. 2015) and the transfer of happiness from humans to adult dogs (D’Aniello et al. 2018) suggest the effects of these chemosignals are relatively stable. The absence of a distinct behavior repertoire in the case of the puppies suggests that the puppies are not biologically prepared to process happiness odors. It is possible to argue, along the lines suggested by Maynard Smith and Harper (2003), that the happiness chemosignal was produced incidentally in response to specific external events. Thus, over time, the released chemosignals can become cues for others to pick up and use. The cue rewarded the herd and evolved to do so, thus transforming the cue into a chemosignal. Therefore, a beneficial characteristic repeatedly experienced by ‘parents’ can have become part of what was inherited (Heyes et al. 2020). As the original Baldwin (Baldwin 1896) argument goes, learning products are likely to have become inherited genetically. The counterpoint for happiness is that it is learned during socialization, whereby happiness chemosignals do not constitute evolutionarily conserved chemosensory cues. This conclusion is strengthened when one considers puppies' social interactions during early infancy. As already specified in the introduction, puppies are more likely to be exposed to human happiness in a family environment. So, if learning and generalization were responsible for rapidly acquiring behavioral patterns in response to chemically driven happiness cues, then one would expect the reverse of the findings we observed in our study. Nevertheless, the a priori and predicted instance-based classifications for the happiness odor condition reveal a relatively low percentage for the happiness condition even in the adult dog condition (53.8%) and is very low in the case of the puppies (40%). These figures suggest that the stability and high percentages in the case of fear odor condition classifications contrast strongly with the happiness case suggesting that even for adult dogs, happiness odor does not have as stable effects as fear odor.

Are puppies unable to detect happiness chemosignals, or are they simply not expressing happiness-compatible behaviors? In other words, can puppies possibly recognize the emotional states induced by happiness odors but do not possess a distinctive behavioral repertoire that differs systematically from a control odor? Our protocol does not allow disentangling this suggestion, which requires a different experimental paradigm. Considerations such as these invite the introduction of new observational paradigms and puppy-compatible psychophysiological measures. However, even if puppies could detect happiness chemosignals, they would still need to learn the appropriate behavioral repertoire. This again underlines the significance of ontogenetic learning for happiness chemical cues.

The effect of the fear chemosignals requires a more careful examination. As argued in the introduction, fear odor perception is under strong selective pressure, and fear is a response observed across animal species and is conserved in mammal brains (LeDoux 2012). Behavioral effects of the fear referred to as “innate fear” (Blanchard and Blanchard 1989) have been observed in rodents as a response to some odors (Stowers and Kuo 2015). Odorants from bodily secretions of predators (Isogai et al. 2011; Samuel et al. 2020) and odors from stressed conspecifics (Brechbühl et al. 2008; Rosen et al. 2015) elicit innate fear responses in their recipients. On the other hand, preparedness theory predicts that responses to some signals are biologically prepared, such as fear signals, facilitating learning owing to their high relevance for survival (Seligman 1971). Accordingly, it is possible that puppies are preconfigured for human fear chemosignals. Therefore, one could surmise that the findings indicate is that dogs' behavioral response to human fear chemosignals is an evolutionarily preconfigured response to a chemosensory cue. Arshamian et al. (2017) showed that some chemical signals are highly conserved, triggering both approach and avoidance (fear) in a predator–prey-predicted manner across taxonomically distant species. Moreover, Iravani et al. (2021) suggested that one of the initial evolutionary functions of olfactory sensory receptivity is rapidly processing and extracting unpleasant odor-based warning signals to modulate approach-avoidance responses. Thus, the response pattern we noted with fear chemosignals is likely to have been shaped long before domestication. This, however, remains a highly tentative conclusion.

The different effects of happiness and fear chemosignals are not surprising, considering previous findings demonstrating that pleasant and unpleasant odors are processed fundamentally differently both as a function of age and at the sensorial level. Indeed, a study with humans revealed that the hedonic appreciation of pleasant odors (but not unpleasant ones) could be modulated by age (Joussain et al. 2013). Furthermore, response times for unpleasant odors are significantly shorter than for pleasant odors (Bensafi et al. 2002).

﻿This current comparative study between adult dogs and puppies is a key to unlocking future insights that could lead to a more detailed understanding of social acquisition processes and contrasting these in the context of preconfigured behavioral repertoires activated by evolutionarily conserved chemosensory signals. An important variable that remains an open question is if there are sex differences between male and female puppies. We know from our earlier research (D’Aniello, et al. 2021) that adult female dogs revealed a significantly longer door-directed behavior when exposed to fear odor compared to the control odor condition. Examining whether fear odor manifests sexual dimorphism in ‘escape’ behaviors in the case of puppies is an issue that needs to be addressed in future research. Finally, a further possible future implication of this work is what it means for intraspecies communication for humans via emotion chemosignals. Early emotion chemosignal-driven communication in humans has not been studied, except for in the case of chemosignal produced by mothers' mammae glands, which have been documented to guide a neonatal behavioral and motivational repertoire, essential for survival (Schaal 2010). Investigating neonate and early infant human receptiveness to emotion chemosignals would be an important line of investigation that has hitherto not been undertaken. This would also advance our understanding of the processes of intraspecies and interspecies chemosignal-driven communication and its limits.

## Data Availability

The data for the reported studies can be obtained from Biagio D’Aniello at biagio.daniello@unina.it.
